# Beef Cattle Farmers’ Knowledge, Attitudes, and Practices Toward On-Farm Biosecurity, Antimicrobial Use, and Antimicrobial Resistance in Illinois, United States of America

**DOI:** 10.3390/antibiotics14030282

**Published:** 2025-03-09

**Authors:** Rima Shrestha, Mohammad Nasim Sohail, Csaba Varga

**Affiliations:** 1 Department of Internal Medicine, University of Illinois College of Medicine Peoria, Peoria, IL 61605, USA; rdshrest@uic.edu; 2 Department of Pathobiology, College of Veterinary Medicine, University of Illinois Urbana-Champaign, Urbana, IL 61802, USA; mnsohail@illinois.edu; 3 Carl R. Woese Institute for Genomic Biology, University of Illinois Urbana-Champaign, Urbana, IL 61802, USA

**Keywords:** antimicrobial use, antimicrobial resistance, farm biosecurity, beef cattle, Illinois, United States, knowledge, attitudes, practices, survey

## Abstract

**Background/Objectives**: Understanding beef cattle farmers’ knowledge, attitudes, and practices on infectious disease prevention, antimicrobial use, and antimicrobial resistance (AMR) is important to developing stewardship programs. **Methods**: A cross-sectional stratified mail or phone survey of beef cattle producers in Illinois was conducted between June and August 2022. Ordinal logistic regression models assessed the impact of having a biosecurity plan on beef cattle farmers’ familiarity with cattle diseases. Logistic regression models evaluated associations between antimicrobial treatment practices and the type of cattle operations. **Results**: A total of 514 producers responded to all or some of the questions. Only 45% of producers were familiar with AMR, and 11% were concerned about cattle infections with antibiotic-resistant bacteria. Producers agreed or strongly agreed (64%) that inappropriate AMU contributes to the development of AMR. Most producers (70%) thought that antimicrobials were as effective in treating infectious diseases as 5 years ago. Only 50% of farms were visited by a veterinarian in the previous year and 35% had their biosecurity evaluated. Producers were more familiar with infectious diseases if their farm biosecurity was assessed. Treating respiratory infections was the most common reason for antimicrobial use. Compared to cow–calf farmers, whole-cycle farmers had a higher probability of having their farm’s biosecurity evaluated (OR = 1.66) and having a veterinarian visit in the previous year (OR = 2.16). Whole-cycle (OR = 3.92) and stocker/backgrounder (OR = 2.18) farmers had a higher probability of treating their cattle with antibiotics than cow–calf farmers. **Conclusions**: Antimicrobial stewardship and farm biosecurity programs are needed to raise awareness of disease prevention, AMU, and AMR among Illinois beef cattle producers.

## 1. Introduction

In the United States of America (US), the beef cattle industry is an important contributor to the local agricultural economy and food supply. The US is also one of the largest beef cattle producers and consumers and ranks second beef exporter worldwide [1]. As of 1 January 2024, Illinois farmers have raised an estimated 331,000 beef cows, ranking Illinois ninth in the U.S. for beef cow inventory [2]. The U.S. beef industry includes different production types, including cow–calf operations, stocker operations, feedlots, and whole-cycle operations that include all production types, and each segment differs in its disease prevention and antimicrobial use practices.

Cow–calf operations are the most common beef production type, where calves are born and raised until they are weaned and sold. Antimicrobials are used in these farms to treat infectious diseases, mainly in young animals, and infections related to the reproductive tract in cows [3]. Stocker operations, which raise weaned calves before entering feedlots, use antimicrobials to manage respiratory diseases and other health issues triggered by high-stress periods of weaning and transportation and mixing calves from different sources [4]. Feedlots, where cattle are finished on a diet designed to maximize weight gain before slaughter, face infectious disease challenges, such as respiratory and gastrointestinal infections, due to the high density of animals and mixing of animals. To control these infections, antimicrobials are commonly used [5].

Effective biosecurity practices on beef cattle farms can prevent the introduction and spread of infectious diseases within and between farms [6]. Reducing the pathogen load on farms might decrease antimicrobial use [7,8]. Also, beef cattle producers who have adopted biosecurity measures on their farms might correlate with their improved understanding of disease prevention and management. In addition, implementing biosecurity protocols on beef cattle farms might influence farmers’ ability to identify potential disease risks and respond effectively to outbreaks. However, biosecurity and antimicrobial use practices might differ among beef cattle operations and be affected by farm size, production type, farm management practices, and producer knowledge [9].

Antimicrobial use (AMU) in beef cattle farms is essential for maintaining optimal health by treating bacterial infectious diseases. However, the overuse and misuse of antimicrobials in food animals have been described as one of the main contributors to the selection of antimicrobial-resistant bacteria [10,11,12]. There is a concern over the development of antimicrobial resistance (AMR) in beef cattle farms, which reduces the effectiveness of antimicrobials in treating infections and increases treatment costs, morbidity, and mortality [13,14]. In addition, antimicrobial-resistant pathogens can enter the food supply and infect humans, posing a public health challenge [15].

In the U.S., to reduce the use of antimicrobials and combat the selection of AMR, the U.S. Food and Drug Administration (FDA) introduced measures to reduce the unnecessary use of antimicrobials in livestock and removed growth promotion as an approved use of medically important antimicrobials [16]. In addition, in-feed and in-water use of medically important antimicrobials requires veterinary oversight, and these antimicrobials can only be administered under the supervision of a licensed veterinarian [17].

The role of beef cattle farmers in following these regulations and their understanding of farm biosecurity, AMU, and AMR is important to the success of these measures [18,19]. However, studies assessing beef cattle farmers’ knowledge, attitudes, and practices toward AMU and AMR issues are lacking in Illinois. Understanding beef cattle farmers’ perspectives on cattle diseases, AMR, and AMU is important to develop effective antimicrobial stewardship programs. Also, identifying gaps in knowledge and assessing whether current practices align with best-practice recommendations will aid in pathogen reduction in cattle farms and reduce the emergence of AMR.

Considering all the issues presented above, this study administered a survey to beef cattle farmers in Illinois to (1) assess their knowledge, attitudes, and practices regarding AMU and AMR; (2) evaluate the impact of having a biosecurity plan on beef cattle farmers’ familiarity with cattle diseases; and (3) assess differences in disease prevention and AMU practices among beef cattle production types.

## 2. Results

### 2.1. Descriptive Analysis

Of the 3000 surveys sent to the beef cattle producers in Illinois between June and August 2022, 514 producers (17.13%) responded to all or some of the questions.

#### 2.1.1. Knowledge and Attitudes on Antimicrobial Resistance and Antimicrobial Use

**Figure 1:**
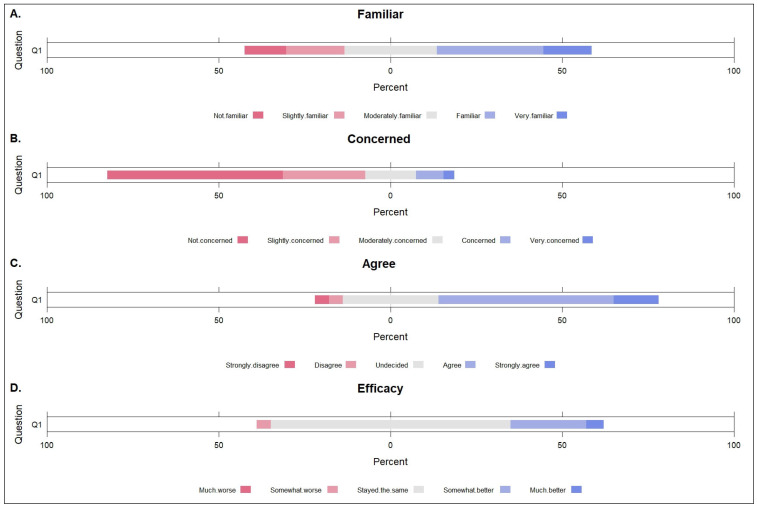
Beef cattle farmers’ responses to questions on their knowledge and attitudes on antimicrobial resistance and antimicrobial use, Illinois, United States. (**A**). How familiar are you with the topic of antibiotic resistance? (**B**). Are you concerned about cattle infections with antibiotic-resistant bacteria on your farm? (**C**). Do you agree that inappropriate antimicrobial use can contribute to the development of antibiotic-resistant bacteria on your farm? (**D**). How do you think antibiotic drugs are working on your farm today compared to 5 years ago?

About half of the beef cattle farmers knew about antibiotic resistance issues (31% familiar and 14% very familiar) (Figure 1).

Half of the producers (51%) were not concerned about cattle infections with antibiotic-resistant bacteria on their farms, whereas 24% were slightly concerned, 15% moderately concerned, 8% concerned, and 3% were very concerned (Figure 1).

Similarly, most beef cattle producers agreed (51%) and strongly agreed (13%) with the statement, “Inappropriate antimicrobial use can contribute to the development of antibiotic-resistant bacteria on your farm.” However, some producers were undecided (28%), disagreed (4%), or strongly disagreed (4%).

When producers were asked how antibiotic drugs are effective on their farms today compared to 5 years ago, 70% responded that they stayed the same (Figure 1). In contrast, 4% responded that the drug’s efficacy somewhat worsened, 22% thought it was somewhat better, and 5% thought it worked much better.

#### 2.1.2. Disease Prevention and Antimicrobial Treatment Practices

Fifty percent of beef cattle producers in Illinois mentioned veterinarian visits to their farms during the previous year. However, only 35% of cattle producers conducted a disease prevention and control (i.e., biosecurity) evaluation on their farms.

**Figure 2:**
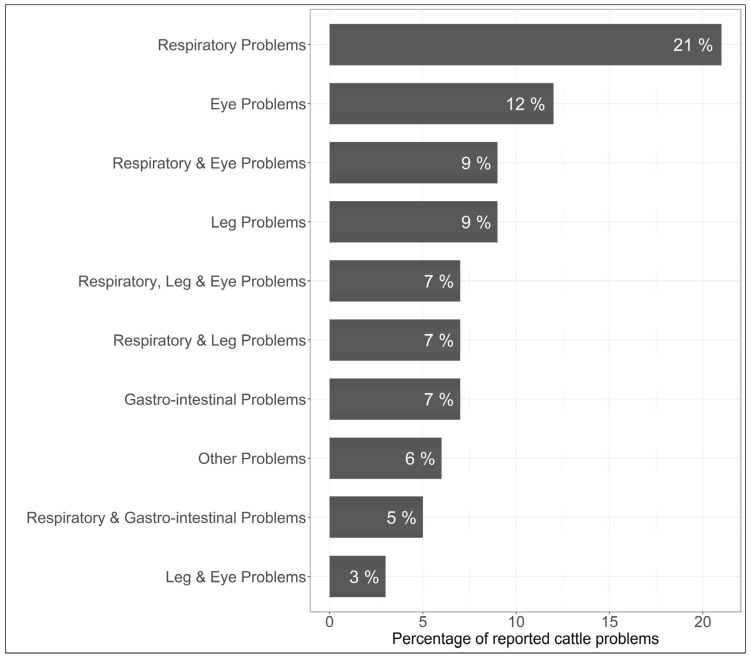
Answers to questions on the conditions for which antibiotics were used in beef cattle farms in Illinois, US.

The beef cattle producers also responded to questions on antimicrobial use practices on their farms (Figure 2).

The largest proportion of farms used antimicrobials to treat respiratory problems (21%), followed by eye problems (12%), leg problems (9%), and gastrointestinal problems (7%). Also, antimicrobials were used for multiple conditions to treat respiratory and leg problems (9%), respiratory and eye problems (7%), and respiratory, eye, and leg problems (7%) (Figure 2).

### 2.2. Regression Models

#### 2.2.1. Familiarity with Cattle Diseases

Beef cattle producers who conducted biosecurity evaluations on their farms were highly knowledgeable about various cattle diseases. A result of the univariable ordinal logistic regression model on the impact of farm biosecurity evaluations (predictor variable) on cattle producers’ familiarity with various infectious diseases (4-scale outcome) is presented in Table 1.

For all diseases, regardless of farm production type, for producers that had a biosecurity evaluation on their farms, the probability of being in a higher level of the outcome (e.g., familiarity with a disease from “Never heard of it” to “Recognize the name” or from “Know some basics” to “Knowledgeable”) were greater (1.5–2.29 times) than the odds of staying in the current or lower categories (Table 1).

**Figure 3:**
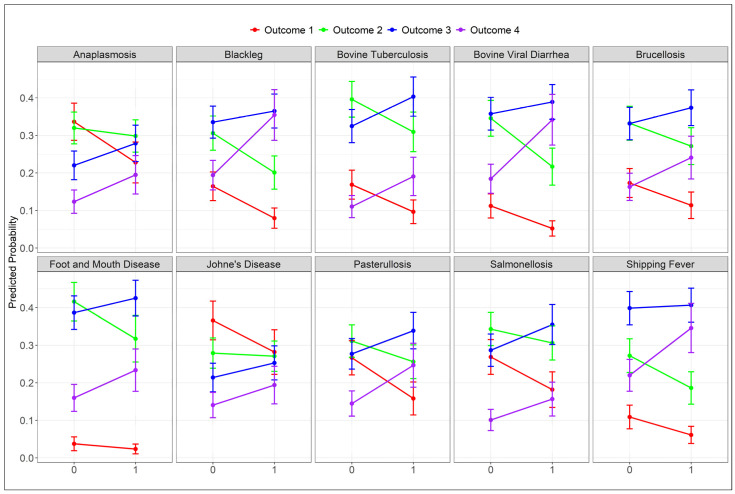
Predicted probabilities of the association between having a biosecurity evaluation on a farm and the beef cattle producers’ familiarity with cattle diseases: The *X*-axis shows having (1) and not having (0) a biosecurity evaluation. The *Y*-axis shows predicted probabilities. Color dots represent the likelihood values of being in each category of the outcomes (1—never heard of it (red); 2—recognize the name (green); 3—know some basics (blue); 4—knowledgeable (purple). The error bars in each outcome represent the 95% confidence intervals for those values.

The predicted probabilities for each level of the outcome (familiarity with a disease on a 4-scale: 1—never heard of it; 2—recognize the name; 3—know some basics; 4—knowledgeable) for the predictor variable (having a biosecurity evaluation (yes = 1) or not (no = 0) on a beef cattle farm) is presented in Figure 3.

For most cattle diseases, beef producers who had their farms’ biosecurity evaluated had a higher predicted probability for the top two outcome categories (category 3— knowing some basics and 4—being knowledgeable about a disease). On the other hand, a higher probability was observed for the two lower outcome categories (Outcomes 1—never heard of it; 2— recognize the name of a disease) for producers who did not conduct a farm biosecurity evaluation on their farm.

#### 2.2.2. Disease Prevention and Antimicrobial Use Practices by Production Type

The results of the univariable logistic regression models for the significant associations in the disease prevention and treatment practices (outcome variable) among the beef cattle production types (predictor variable) in Illinois are presented in Table 2. Cow–calf operations, the most common production type, were included as the referent category.

Whole-cycle beef cattle farmers had a higher probability of a veterinarian visiting their farm in the previous year and having their farm’s biosecurity evaluated than cow–calf operations. The odds of antimicrobial use (AMU) and the use of in-feed antimicrobials were higher on the whole-cycle and in backgrounder/stocker operations than in cow–calf operations. The probability of using antimicrobials in water was the highest in feedlots compared to cow–calf farms. Whole-cycle farms had higher odds of using antimicrobials to treat gastrointestinal problems than cow–calf farms. Compared to cow–calf operations, all the other production types had a higher probability of using antimicrobials to treat respiratory problems. Feedlots and backgrounder/stocker operations had higher odds of using antimicrobials to treat eye problems than cow–calf farms. No significant differences were observed among beef cattle production types and the use of antimicrobials as injections or to treat reproductive problems, liver abscesses, and feet/leg problems.

## 3. Discussion

This study used a cross-sectional survey to assess the current knowledge, attitudes, and practices related to AMU, AMR, and disease prevention among beef cattle producers in Illinois, U.S. Differences in AMU practices and disease prevention were found across different beef cattle production systems. Whole-cycle production type farms compared to cow–calf had a higher probability of having a veterinarian visit the previous year and using antimicrobials to treat cattle diseases, including respiratory and gastrointestinal conditions. Beef cattle farmers who had their farm biosecurity evaluated had a higher probability of being familiar with cattle diseases. We identified current knowledge and practices related to disease prevention and AMU that can aid animal health stakeholders in developing antimicrobial stewardship and outreach programs.

Around half of the respondents were familiar with the issue of AMR, but their degree of concern about cattle infections with antibiotic-resistant bacteria varied. A gap between familiarity with AMR and awareness and concern about infection with antimicrobial-resistant bacteria was observed among Illinois beef cattle producers. While 45% of producers were at least somewhat concerned, 51% of producers were not concerned at all. This finding is consistent with a previous study from Tennessee, U.S., which described that, while many producers were aware of the issue of AMR, they lacked an understanding of the negative implications of AMR on cattle health and public health [20].

It is well documented that AMU can impact the selection of AMR in beef cattle farms [13,21]. In our study, over half of the producers agreed that inappropriate antimicrobial use contributes to the development of AMR, but 28% of respondents were undecided or disagreed, indicating that knowledge gaps persist among Illinois producers, which might hinder responsible AMU practices. This knowledge gap might be explained by beef farmers’ lack of up-to-date, accessible educational resources on AMR and AMU. Also, in economically disadvantaged areas, farmers may not have access to veterinarians with expertise in antimicrobial stewardship [9]. This lack of guidance may prevent farmers from learning about proper AMU and alternatives to antibiotic treatments, including biosecurity methods that can reduce pathogens on farms [6].

Most producers felt that the efficacy of antimicrobials remained stable over the past five years. While a few producers reported a decrease in efficacy, the majority reported no change, suggesting that the effects of AMR on individual farms are not perceived as important. This finding corresponds with previous studies, which described that livestock producers are hesitant to change AMU practices unless the consequences of AMR are directly observable on their farms [22,23]. Focused education and outreach programs on AMR and AMU are needed to help producers understand the long-term risks of AMR and the need for proactive antimicrobial stewardship.

Differences in Illinois beef cattle producers’ farm biosecurity practices and disease prevention strategies were observed across different production systems. Approximately 50% of producers reported veterinarian visits in the past year, but only 35% conducted a biosecurity evaluation on their farms. Previous research has similarly highlighted the challenges livestock farmers face to obtain access to veterinary care and also described that beef cattle producers, mainly smaller operations, face barriers in implementing biosecurity programs, often due to their limited financial resources [24,25]. Our results emphasize the need for rural livestock veterinarians who can help beef cattle producers integrate biosecurity evaluations as part of their routine herd health management [26,27]. In addition, biosecurity evaluations may include on-site visits by veterinarians or agricultural extension agents, providing farmers with information on disease risks and effective prevention strategies. On beef cattle farms, preventing and controlling the bovine respiratory disease complex (BRD), also known as shipping fever pneumonia, is challenging and frequently involves treating cattle with broad-spectrum antimicrobials, such as macrolides and phenicols. However, farmers can limit prophylactic antimicrobial treatments by implementing effective biosecurity procedures, including quarantining newly introduced cattle, vaccinating animals, reducing cattle stress, monitoring environmental conditions, and providing proper nutrition.

The high prevalence of AMU for treating respiratory, eye, and gastrointestinal problems reflects trends observed in previous studies, which identified respiratory diseases as the primary reason for antimicrobial administration in beef cattle farms [13,28]. In addition, using antimicrobials to treat multiple conditions (e.g., respiratory and eye problems) indicates the complexity of health management on beef farms. However, by identifying disease conditions with high AMU, targeted disease prevention interventions could be implemented to reduce the pathogen load and, consequently, the unnecessary use of antimicrobials [3].

The regression analysis indicated that producers who conducted biosecurity evaluations on their farms were more familiar with cattle diseases, implying that farm biosecurity evaluations are linked with increased awareness and knowledge about disease risks and lower disease rates. This finding agrees with a previous study where regular health assessments and biosecurity measures on cattle farms were linked to a higher likelihood of Bovine Viral Diarrhea-free status [29]. Considering this finding, encouraging producers to have biosecurity evaluations on their farms and improving their knowledge of infectious diseases and associated risks might be beneficial.

We also investigated the differences in disease prevention and antimicrobial use practices across different beef cattle production types. The whole-cycle and feedlot operations had higher veterinarian involvement and biosecurity evaluations than cow–calf operations. This is consistent with previous studies suggesting that larger, more intensive production systems are more likely to implement disease management and biosecurity practices than small-scale farms [30]. Additionally, the higher odds of antimicrobial use for gastrointestinal and respiratory problems in feedlots and backgrounder/stocker operations reflect the higher density of animals, the impact of weather conditions, exposure to environmental stressors during transport and after arrival, and mixing animals from different sources in these production systems, which all predispose cattle to infections that require antimicrobial treatments [31,32,33].

Compared to cow–calf operations, a higher probability of using antimicrobials in feed in the whole-cycle, backgrounder operations, and water in feedlots were observed. This finding is consistent with the industry standards [4], which consider the ease of treatment in a high cattle number and density setting, reflecting the use of antimicrobials to prevent diseases at the whole herd level rather than treating individual animals [34].

Comparing the findings of this study with the regional trends of AMU and AMR is critical to understanding whether our results are Illinois-specific or show the patterns of the U.S. beef cattle industry. In the US, a ban on antimicrobials for growth promotion was implemented in 2017, and also the therapeutic in feed and water use of medically important antimicrobials is only allowed under a veterinarian’s supervision. However, the therapeutic use of antibiotics is a concern, and beef producers rely on antibiotics to manage diseases such as BRD, which is the main reason for morbidity and mortality in feedlots. Illinois is a mid-sized cattle producer state with several small family-owned cow–calf operations and a limited number of larger commercial feedlots. The AMU trends in Illinois beef farms might not reflect the AMU of other larger beef production states across the U.S., which might have higher AMU rates. In addition, the knowledge of Illinois beef cattle producers on AMU and AMR might be lower than in other large cattle producer states because larger operations might have more resources to implement biosecurity protocols, monitor cattle health, and participate in antibiotic stewardship programs.

Before extrapolating our study results, we need to mention a few limitations. The response rate of 17% of the survey is considered acceptable. However, the non-response bias should be considered because responders might differ systematically from non-responders, and farmers who are more familiar with biosecurity practices or have better knowledge of AMU and AMR might be more likely to respond to the survey, which could lead to an overestimation. In addition, addressing potential confounding factors such as farm size, geographical location, veterinary support, economic conditions, and animal health history is necessary to ensure the validity of this study’s conclusions. To address some of these factors, we used stratified random sampling and applied survey weighting that accounted for the farm size, the farm number in each farm size category, and the non-response rate. Finally, this study cannot prove the causality and directionality of the relationship between biosecurity evaluations and improved disease knowledge because we cannot be certain that biosecurity evaluations directly cause improved knowledge or whether more knowledgeable producers are more likely to conduct biosecurity evaluations. A future longitudinal study should be conducted wherein, after a farm biosecurity evaluation and stewardship program implementation, producers would be followed over time, and changes in their knowledge and attitudes would be monitored.

Based on our study results, there is a need for education and outreach to beef cattle producers regarding the risks of AMR and the benefits of improved biosecurity practices for disease prevention. Encouraging farmers to conduct biosecurity evaluations could help with disease prevention, reduce the pathogen load, and consequently reduce the reliance on antimicrobials to treat infectious diseases.

Future research should focus on evaluating the effectiveness of specific biosecurity interventions in reducing pathogens and the need for antimicrobials. Also, assessing longitudinal AMR trends in cattle pathogens and tracking changes in AMU and AMR over time in response to educational programs or policy changes could evaluate the impact of these efforts.

## 4. Materials and Methods

### 4.1. Sampling Frame and Questionnaire Development

The study team developed the questionnaire consulting the existing literature on knowledge, attitudes, and practices toward antimicrobial resistance (AMR) and antimicrobial use (AMU) in beef cattle production in the United States [6,9,20]. The questionnaire was pretested by the study team and was also reviewed by a beef cattle farmer to identify any unclear or ambiguous questions that could confuse respondents. Also, the study team evaluated the overall flow and structure of the survey, estimated the survey completion time, and refined the survey instrument if needed.

The target population included all beef cattle producers in Illinois that were available in the USDA National Agricultural Statistics Service (NASS) database. A sample size of at least 375 respondents was necessary to estimate the proportion of responses to a question (*p* = 0.5) of a small finite population (n = 14,641) with a 5% margin of error and a 95% confidence interval. The sampling frame was stratified based on the farm sizes and the number of farms in each stratum into 4 strata, and survey weighting was applied to the response data. We estimated a 15% response rate, and to achieve the required sample size, the NASS personnel mailed 3000 surveys to Illinois beef cattle producers. The first mailing was on 6 July 2022, the second on 27 July 2022, and the third on 15 August 2022. Non-respondents were followed up by phone calls, and if the producer agreed, the survey was administered via phone.

The survey questionnaire for this study included questions that asked beef cattle farmers in Illinois about their (1) knowledge and attitudes toward AMR and AMU, (2) familiarity with cattle infectious diseases, and (3) disease prevention and treatment practices. Table 3 describes the questions and their type used in this study.

In addition, a predictor variable was constructed that represented the type of beef cattle operation: cow–calf, feedlot, stocker/backgrounder, and “whole-cycle”. The cow-calf operation is a farm wherein cattle are raised on pasture and are kept to breed and raise calves, which are then sold to a stocker/backgrounder operation, a feedlot, or other markets when they are six-to-ten months old. The stocker/backgrounder operation is an extensive beef cattle production phase wherein weaned steer and heifer calves or yearlings are grazed on grass or other roughage and are usually sold to feedlots for further finishing when they are around 12 months of age and approximately weigh 400 kg. The feedlot is the final stage of the beef cattle production chain, wherein cattle are fed grain or other concentrate feeds and grow to slaughter weight (approximately 545 kg), usually when they are 18–22 months old. The “whole-cycle” operation includes all stages of the beef cattle production chain (cow–calf, stocker/backgrounder, and feedlots).

### 4.2. Statistical Analysis

For data analysis, we used STATA Intercooled software (Version 17, Stata Corporation, College Station, TX, USA) and R software in R Studio Platform (Version 1.4.1106 2009–2021 RStudio, PBC) for data visualization using the Likert [35] and ggplot2 [36] packages.

For the descriptive analysis, weighted proportions were calculated for each categorical variable and were illustrated in graphs. Analytical weights were used that considered the farm sizes (Stratum 1: 1 to 76 head of cattle, Stratum 2: 77 to 221 head of cattle, Stratum 3: 222 to 1787 head of cattle, Stratum 4: 1788 or more head of cattle), the number of farms in each stratum (Stratum 1: 11,752 farms, Stratum 2: 2219 farms, Stratum 3: 652 farms, and Stratum 4: 18 farms), and the non-response weights (Straum 1: 3.344, Stratum 2: 4.252, Stratum 3: 5.134, and Stratum 4: 6.000)

#### Regression Models

First, univariable ordinal logistic regression models (one for each cattle disease) were constructed to evaluate associations between the familiarity of the cattle producer with a cattle disease (outcome variable) and having a biosecurity evaluation on the cattle farm (predictor variable). For each model, marginal effects for the predictor variable were calculated, and the predicted probabilities of having a biosecurity evaluation on a farm at each level of the outcome variable (4 levels) were illustrated in line graphs.

Next, univariable logistic regression models were constructed to assess associations between the outcome variable representing having a biosecurity evaluation (model 1), and a veterinarian visiting the farm in the previous year (model 2). In both models, the predictor variable was signified by cattle operation type, including cow–calf operations as the referent category to which the other operation types were compared.

Lastly, univariable logistic regression models were constructed to assess the associations between each antimicrobial treatment practice (outcome variable) and the cattle operation type (predictor variable), including cow–calf operations as the referent category to which the other operation types were compared. The significance of the associations for each model was set to *p*-value ≤ 0.05.

## Figures and Tables

**Table 1:** Results on the impact of farm biosecurity evaluations on producers’ familiarity with various cattle diseases.

Familiarity with a Disease (Outcome) ^a^	Odds Ratio (95% CI ^b^)	*p*-Value ^c^
Brucellosis	1.63 (1.15–2.30)	0.005
Foot and mouth disease	1.60 (1.13–2.28)	0.008
Bovine Viral Diarrhea	2.29 (1.61–3.27)	<0.001
Johne’s disease	1.47 (1.05–2.06)	0.025
Salmonellosis	1.65 (1.17–2.33)	0.004
Bovine tuberculosis	1.90 (1.34–2.70)	<0.001
Bovine respiratory disease complex	1.87 (1.33–2.65)	<0.001
Clostridium Disease (Blackleg)	2.28 (1.61–3.23)	<0.001
Pasteurella	1.94 (1.37–2.74)	<0.001
Anaplasmosis	1.72 (1.22–2.42)	0.002

^a^ Ordinal logistic regression models constructed for each outcome. ^b^ CI: Confidence Interval; ^c^ Significant if *p*-value is ≤0.05.

**Table 2:** Univariable logistic regression models on the associations between disease prevention and antimicrobial use practices and beef cattle production types in Illinois, U.S.

Outcome Variable	Predictor Variable	Odds Ratio (95% CI)	*p*-Value
Having a farm biosecurity evaluation	Cow–calf (Referent)	-	-
	Feedlot	0.99 (0.54–1.83)	0.983
	Backgrounder/Stocker	1.20 (0.66–2.19)	0.551
	Whole-cycle	1.66 (1.05–2.63)	0.031
A veterinarian visited the farm in the previous year	Cow–calf (Referent)	-	-
	Feedlot	0.61 (0.34–1.10)	0.103
	Backgrounder/Stocker	1.14 (0.62–2.09)	0.676
	Whole-cycle	2.16 (1.28–3.64)	0.004
Cattle were treated with antimicrobials	Cow–calf (Referent)	-	-
	Feedlot	1.00 (0.55–1.81)	0.995
	Backgrounder/Stocker	2.18 (1.12–4.25)	0.022
	Whole-cycle	3.92 (2.16–7.13)	<0.001
Antimicrobials were used in feed	Cow–calf (Referent)	-	-
	Feedlot	1.22 (0.52–2.85)	0.649
	Backgrounder/Stocker	2.20 (1.08–4.50)	0.031
	Whole-cycle	1.93 (1.10–3.36)	0.021
Antimicrobials were used in water	Cow–calf (Referent)	-	-
	Feedlot	4.95 (1.58–15.46)	0.006
	Backgrounder/Stocker	2.86 (0.89–9.27)	0.079
	Whole-cycle	1.31 (0.41–4.12)	0.649
Antimicrobials were used to treat gastrointestinal problems	Cow–calf (Referent)	-	-
	Feedlot	0.68 (0.26–1.76)	0.423
	Backgrounder/Stocker	1.63 (0.79–3.35)	0.185
	Whole-cycle	1.83 (1.05–3.18)	0.033
Antimicrobials were used to treat respiratory problems	Cow–calf (Referent)	-	-
	Feedlot	4.23 (1.55–11.52)	0.005
	Backgrounder/Stocker	3.95 (1.66–9.39)	0.002
	Whole-cycle	2.82 (1.56–5.10)	0.001
Antimicrobials were used to treat eye problems	Cow–calf (Referent)	-	-
	Feedlot	0.27 (0.10–0.73)	0.010
	Backgrounder/Stocker	2.17 (1.08–4.36)	0.029
	Whole-cycle	0.73 (0.43–1.26)	0.260

**Table 3:** Question types assessing beef cattle farmers’ knowledge, attitudes, and practices toward biosecurity, antimicrobial resistance, and antimicrobial use.

Question Types	Answers	Measurements
Knowledge and Attitudes on AMR and AMU *		
How familiar are you with the topic of antibiotic resistance?	Not familiar/Slightly familiar/Moderately familiar/Familiar/Very familiar	Ordinal (5-scale)
Are you concerned about cattle infections with antibiotic-resistant bacteria on your farm?	Not concerned/Slightly concerned/Moderately concerned/Concerned/Very concerned	Ordinal (5-scale)
Do you agree that inappropriate antimicrobial use can contribute to the development of antibiotic-resistant bacteria on your farm?	Strongly disagree/Disagree/Undecided/Agree/Strongly agree	Ordinal (5-scale)
How do you think antibiotic drugs are working on your farm today compared to 5 years ago?	Much worse/Somewhat worse/Stayed the same/Somewhat better/Much better	Ordinal (5-scale)
**Familiarity with cattle diseases**		
Please describe your familiarity with the following diseases: Brucellosis, Anthrax, Foot-and-mouth disease (FMD), Bovine Viral Diarrhea (BVD), Johne’s disease, Salmonellosis, Bovine Tuberculosis (TB), Bovine respiratory disease complex (Shipping fever), Clostridium Disease (Blackleg), Pasteurella, Anaplasmosis (Yellow fever).	Never heard of it/Recognize the name/Know some basics/Knowledgeable	Ordinal (4-scale)
**Disease Prevention and Treatment Practices**		
Has your cattle operation conducted a disease prevention and control (i.e., biosecurity) evaluation? (This could be done by yourself, your veterinarian, an extension agent, or other knowledgeable people.)?	Yes/No	Categorical, Binomial
Did a veterinarian visit your farm in 2021 regarding your cattle?	Yes/No	Categorical, Binomial
Did you treat your cattle with antibiotics in 2021?	Yes/No	Categorical, Binomial
If you treated your cattle with antibiotics, what was the administration route? (Check all that apply.)	Water/Feed/Injection	Categorical, Binomial
For which condition(s)/reason(s) were antibiotics used? (Check all that apply)	Gastrointestinal problems (e.g., diarrhea)/Respiratory problems (e.g., pneumonia, cough)/Reproductive problems (e.g., calving problems, uterine infections)/Liver abscesses/Eye problems (e.g., pink eye)/Feet/leg problems (e.g., lameness)	Categorical

* AMR: Antimicrobial resistance; AMU: Antimicrobial use.

## Data Availability

All materials needed to replicate the findings of the article are available upon request from the USDA National Agricultural Statistics Service (NASS).

## Abbreviations

The following abbreviations are used in this manuscript:

AMR	Antimicrobial resistance
AMU	Antimicrobial use
BRD	Bovine respiratory disease complex
NASS	National Agricultural Statistics Service
US	United States of America
USDA	United States Department of Agriculture

## References

[1] USDA Economic Research Service Cattle and Beef—Sector at a Glance. https://www.ers.usda.gov/topics/animal-products/cattle-beef/sector-at-a-glance.

[2] USDA National Agricultural Statistics Service (NASS) (2023). 2023 State Agriculture Overview for Illinois.

[3] Lhermie G., Verteramo Chiu L., Kaniyamattam K., Tauer L.W., Scott H.M., Gröhn Y.T. (2019). Antimicrobial policies in United States beef production: Choosing the right instruments to reduce antimicrobial use and resistance under structural and market constraints. Front. Vet. Sci..

[4] USDA USDA–APHIS–VS–CEAH–NAHMS. Fort Collins, CO. Antimicrobial use and stewardship on U.S. feedlots, 2017. #751.0419. https://www.aphis.usda.gov/sites/default/files/amu-feedlots.pdf.

[5] Apley M.D., Schrag N.F.D., Amrine D.E., Lubbers B.V., Singer R.S. (2023). Antimicrobial use in 20 U.S. beef feedyards: 2018–2019. Front. Vet. Sci..

[6] Pudenz C.C., Mitchell J.L., Schulz L.L., Tonsor G.T. (2021). U.S. Cattle producer adoption of Secure Beef Supply Plan enhanced biosecurity practices and Foot-and-Mouth Disease preparedness. Front. Vet. Sci..

[7] Diana A., Lorenzi V., Penasa M., Magni E., Alborali G.L., Bertocchi L., De Marchi M. (2020). Effect of welfare standards and biosecurity practices on antimicrobial use in beef cattle. Sci. Rep..

[8] Plummer P., Fajt V.R. (2024). Biosecurity practices to enhance responsible antimicrobial use and reduce the burden of antimicrobial resistance. Vet. Clin. N. Am. Food Anim. Pract..

[9] Ekakoro J.E., Caldwell M., Strand E.B., Okafor C.C. (2019). Drivers, alternatives, knowledge, and perceptions towards antimicrobial use among Tennessee beef cattle producers: A qualitative study. BMC Vet. Res..

[10] Shrestha R.D., Agunos A., Gow S.P., Deckert A.E., Varga C. (2022). Associations between antimicrobial resistance in fecal *Escherichia coli* isolates and antimicrobial use in Canadian turkey flocks. Front. Microbiol..

[11] Varga C., Rajić A., McFall M.E., Reid-Smith R.J., Deckert A.E., Checkley S.L., McEwen S.A. (2009). Associations between reported on-farm antimicrobial use practices and observed antimicrobial resistance in generic fecal *Escherichia coli* isolated from Alberta finishing swine farms. Prev. Vet. Med..

[12] Benedict K.M., Gow S.P., McAllister T.A., Booker C.W., Hannon S.J., Checkley S.L., Noyes N.R., Morley P.S. (2015). Antimicrobial resistance in *Escherichia coli* recovered from feedlot cattle and associations with antimicrobial use. PLoS ONE.

[13] Cameron A., McAllister T.A. (2016). Antimicrobial usage and resistance in beef production. J. Anim. Sci. Biotechnol..

[14] Strong K.M., Marasco K.L., Invik J., Ganshorn H., Reid-Smith R.J., Waldner C.L., Otto S.J.G., Kastelic J.P., Checkley S.L. (2023). Factors associated with antimicrobial resistant *Enterococci* in Canadian beef cattle: A scoping review. Front. Vet. Sci..

[15] Stapleton G.S., Innes G.K., Nachman K.E., Casey J.A., Patton A.N., Price L.B., Tartof S.Y., Davis M.F. (2024). Assessing the Difference in contamination of retail meat with multidrug-resistant bacteria using for-consumer package label claims that indicate on-farm antibiotic use practices—United States, 2016–2019. J. Expo. Sci. Environ. Epidemiol..

[16] FDA (2013). Guidance for Industry #213. New Animal Drugs and New Animal Drug Combination Products Administered in or on Medicated Feed or Drinking Water of Food-Producing Animals: Recommendations for Drug Sponsors for Voluntarily Aligning Product Use Conditions with GFI #209. https://www.fda.gov/media/83488/download.

[17] FDA (2015). Veterinary Feed Directive. Federal Register. Rules and Regulations. https://www.govinfo.gov/content/pkg/FR-2015-06-03/pdf/2015-13393.pdf.

[18] Dillon M.E., Jackson-Smith D. (2021). Impact of the veterinary feed directive on Ohio cattle operations. PLoS ONE.

[19] Ekakoro J.E., Caldwell M., Strand E.B., Okafor C.C. (2019). Perceptions of Tennessee cattle producers regarding the Veterinary Feed Directive. PLoS ONE.

[20] Ekakoro J.E., Caldwell M., Strand E.B., Strickland L., Okafor C.C. (2019). A survey of antimicrobial use practices of Tennessee beef producers. BMC Vet. Res..

[21] Doster E., Pinnell L.J., Noyes N.R., Parker J.K., Anderson C.A., Booker C.W., Hannon S.J., McAllister T.A., Gow S.P., Belk K.E. (2022). Evaluating the effects of antimicrobial drug use on the ecology of antimicrobial resistance and microbial Community structure in beef feedlot cattle. Front. Microbiol..

[22] Doidge C., Hudson C.D., Burgess R., Lovatt F., Kaler J. (2020). Antimicrobial use practices and opinions of beef farmers in England and Wales. Vet. Rec..

[23] Thongyuan S., Tansakul N. (2024). Antimicrobial use on pig farms in Thailand: Farmer perceptions of use and resistance. Prev. Vet. Med..

[24] Barnhardt T.R., Raabis S.M. (2024). Biosecurity strategies for optimization of calf health in North American beef and dairy operations. Vet. Clin. N. Am. Food Anim. Pract..

[25] Wennekamp T.R., Waldner C.L., Parker S., Windeyer M.C., Larson K., Campbell J.R. (2021). Biosecurity practices in western Canadian cow-calf herds and their association with animal health. Can. Vet. J..

[26] Howarth B.E., van Winden S. (2021). Changing veterinary attitudes towards delivering biosecurity advice to beef farmers. Animals.

[27] Agrawal I., Varga C. (2024). Assessing and comparing disease prevention knowledge, attitudes, and practices among veterinarians in Illinois, United States of America. Prev. Vet. Med..

[28] Hope K.J., Apley M.D., Schrag N.F.D., Lubbers B.V., Singer R.S. (2020). Antimicrobial use in 22 U.S. beef feedyards: 2016–2017. Zoonoses Public Health.

[29] Renault V., Lomba M., Delooz L., Ribbens S., Humblet M.F., Saegerman C. (2020). Pilot study assessing the possible benefits of a higher level of implementation of biosecurity measures on farm productivity and health status in Belgian cattle farms. Transbound. Emerg. Dis..

[30] Morris G., Ehlers S., Shutske J.U.S. (2023). Small-scale livestock operation approach to biosecurity. Agriculture.

[31] Abi Younes J.N., Campbell J.R., Gow S.P., Woolums A.R., Waldner C.L. (2024). Association between respiratory disease pathogens in calves near feedlot arrival with treatment for bovine respiratory disease and subsequent antimicrobial resistance status. Front. Vet. Sci..

[32] Cernicchiaro N., Renter D.G., White B.J., Babcock A.H., Fox J.T. (2012). Associations between weather conditions during the First 45 days after feedlot arrival and daily respiratory disease risks in autumn-placed feeder cattle in the United States. J. Anim. Sci..

[33] Cernicchiaro N., White B.J., Renter D.G., Babcock A.H., Kelly L., Slattery R. (2012). Effects of body weight loss during transit from sale barns to commercial feedlots on health and performance in feeder cattle cohorts arriving to feedlots from 2000 to 2008. J. Anim. Sci..

[34] Brault S.A., Hannon S.J., Gow S.P., Warr B.N., Withell J., Song J., Williams C.M., Otto S.J.G., Booker C.W., Morley P.S. (2019). Antimicrobial use on 36 beef feedlots in western Canada: 2008–2012. Front. Vet. Sci..

[35] Bryer J., Speerschneider K. (2016). Analysis and visualization Likert items. Compr. R Arch. Netw..

[36] Wickham H. (2016). . Ggplot2: Elegant Graphics for Data Analysis.

